# Hierarchical, Multi-Sensor Based Classification of Daily Life Activities: Comparison with State-of-the-Art Algorithms Using a Benchmark Dataset

**DOI:** 10.1371/journal.pone.0075196

**Published:** 2013-10-09

**Authors:** Heike Leutheuser, Dominik Schuldhaus, Bjoern M. Eskofier

**Affiliations:** Digital Sports Group, Pattern Recognition Lab, Department of Computer Science, Friedrich-Alexander-University Erlangen-Nuremberg, Germany; Rutgers University, United States of America

## Abstract

Insufficient physical activity is the 4th leading risk factor for mortality. Methods for assessing the individual daily life activity (DLA) are of major interest in order to monitor the current health status and to provide feedback about the individual quality of life. The conventional assessment of DLAs with self-reports induces problems like reliability, validity, and sensitivity. The assessment of DLAs with small and light-weight wearable sensors (e.g. inertial measurement units) provides a reliable and objective method. State-of-the-art human physical activity classification systems differ in e.g. the number and kind of sensors, the performed activities, and the sampling rate. Hence, it is difficult to compare newly proposed classification algorithms to existing approaches in literature and no commonly used dataset exists. We generated a publicly available benchmark dataset for the classification of DLAs. Inertial data were recorded with four sensor nodes, each consisting of a triaxial accelerometer and a triaxial gyroscope, placed on wrist, hip, chest, and ankle. Further, we developed a novel, hierarchical, multi-sensor based classification system for the distinction of a large set of DLAs. Our hierarchical classification system reached an overall mean classification rate of 89.6% and was diligently compared to existing state-of-the-art algorithms using our benchmark dataset. For future research, the dataset can be used in the evaluation process of new classification algorithms and could speed up the process of getting the best performing and most appropriate DLA classification system.

## Introduction

According to the World Health Organization, the 4th leading risk factor for mortality is insufficient physical activity [1]. Approximately 3.2 million people of the world population decease each year because of insufficient physical activity [1]. Furthermore, the risk of all-cause mortality is 20% to 30% higher for people with inadequate physical activity compared to those who perform moderate physical activities at least 30 minutes a day [1]. Moderate physical activities are for example walking, ascending stairs or certain household activities. Walking short distances (instead of driving) or ascending stairs (instead of taking an elevator) are modest possibilities to enhance one’s own activity level day by day [2]. Apart from the effect of moderate physical activity regarding all-cause mortality, it is assumed that the participation in 150 minutes of moderate physical activity per week reduces the risk of ischaemic heart disease by approximately 30%, the risk of diabetes by 27%, and the risk of breast and colon cancer by 21% to 25% [1].

Ogden et al. [3] state that the prevalence of overweight among adolescents aged 2 to 19 years and obesity among men increased significantly during 1999 to 2004. They assume that the increase in body weight is continuing in men, adolescents and children. Wing et al. [4] review the evidence regarding the role of physical activity in the treatment of adult overweight and obesity. They conclude that it is of major interest to develop better ways of measuring exercise. Thereby better types of exercise can be defined that will lead to more adherence to exercise and thus long-term weight loss.

A wide range of studies show that physically active people have higher levels of health-related fitness and lower rates of various chronic diseases compared to physically inactive people [5]–[11]. Methods for assessing the individual daily life activity (DLA) are of major interest in order to monitor the current health status and to provide feedback about the individual quality of life.

DLAs can be assessed by different methods. An overview of these methods is given by Warren et al. [12]. Self-reports like questionnaires and activity diaries are a widely used tool to assess physical activity. They provide physical activity data from a large number of people in short time. However, self-reports induce problems with reliability, validity and sensitivity [13]. Therefore, the current trend is to replace self-reports with automatic DLA classification based on small and light-weight wearable sensors like inertial measurement units. These sensors provide a reliable and objective measurement of physical activity.

Mannini and Sabatini [14] provide an overview of state-of-the-art human physical activity classification systems. Most of the approaches used accelerometers but differed in

- number of sensor axes (uniaxial, biaxial and triaxial accelerometer),
- number of sensors and sensor placement,
- sampling rate,
- number of subjects,
- computed features,
- epoch/window size, and
- number and type of activities.

Regarding all these differences, it is difficult to compare newly proposed methods to existing approaches in the literature. Ideally, newly proposed methods are compared with other approaches in the literature based on the same benchmark dataset.

The purpose of this paper is twofold. First, we provide an extensive, publicly available dataset of DLAs to be used as a benchmark for new algorithms in the future (http://www.activitynet.org). Second, we propose a novel, hierarchical, multi-sensor based classification system for DLAs, that is diligently compared to existing systems.

Our assumption was that sensor fusion of accelerometers and recently increasingly available gyroscopes improves the distinction of several single activities like ascending or descending stairs. For data generation, we measured 23 subjects with accelerometer and gyroscope sensors placed at four body positions: wrist, hip, chest and ankle. Thirteen activities were considered including postures (sitting, lying, standing), household activities (washing dishes, vacuuming, sweeping), walking behaviors (normal walking, running, stairs climbing), and sports activities (bicycling, rope jumping). Our proposed classification system consisted of a hierarchical classifier structure that is flexible in its applicability to other activities that were not investigated in the current study. In a first step, one classifier, in the following sections denoted by BASE, was used to distinguish between several activity groups. In a second step, separate classifiers, in the following sections denoted by HOUSE, REST, WALK, and BICYCLE, were used to discriminate between the single activities that were included in each group. In order to compare the proposed approach to existing algorithms, several state-of-the-art approaches in the literature were implemented and evaluated using the provided benchmark dataset.

## Methods

### Hardware Equipment and Sensor Setup

**Figure 1:**
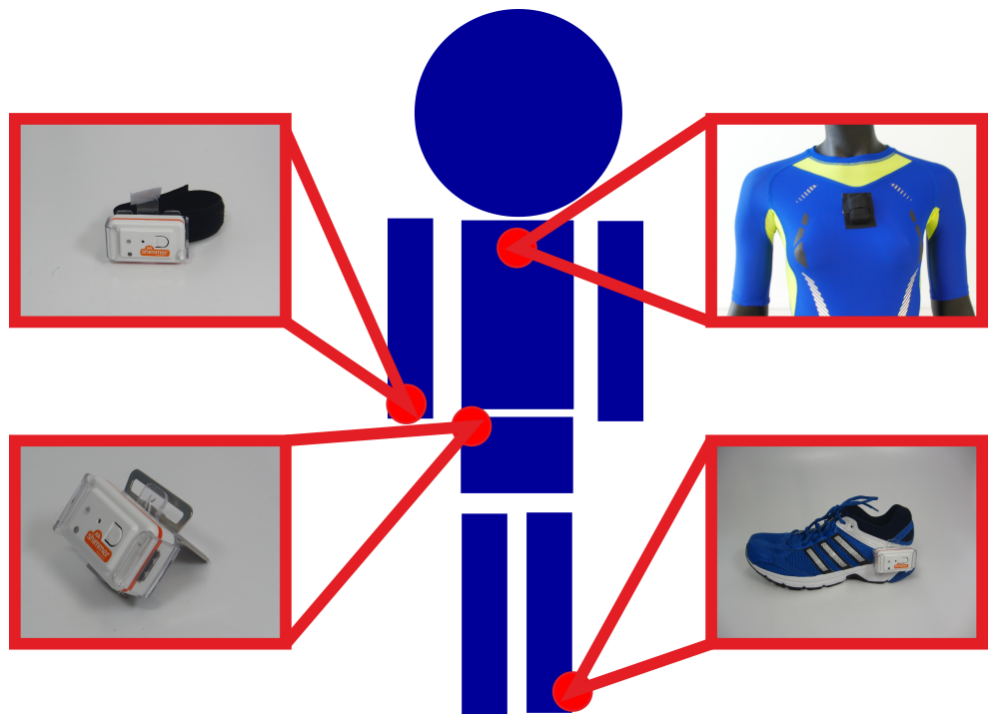
Sensor placement. Four SHIMMER sensor nodes were placed on the wrist, chest, hip, and ankle.

We collected data using four SHIMMER (Shimmer Research, Dublin, Ireland) sensor nodes [15]. The SHIMMER sensor node contains a MSP430F1611 microcontroller. The resolution of the analog-to-digital converter was 12 bit. Each sensor node consisted of three accelerometer and three gyroscope axes. The four sensor nodes were placed on the right hip, the chest, the right wrist, and the left ankle (Fig. 1). These four positions were chosen according to previously published results, which are mentioned in the following.

Sensors closely attached to the bodys center of gravity are to be preferred [16]. Sensors on the chest, the trunk or the hip satisfy this condition. Sensors on the hip are used in a variety of different studies [16]–[22]. Sensors on the trunk or chest are also common in the literature [23], [24]. To cover the extremities, one sensor was placed on the wrist and one on the ankle. Sensors on the wrist enable a correct classification of activities mainly dominated by the upper body [17]–[19], [23]. Positioning a sensor on the ankle is heavily used in gait analysis [25] and activity recognition studies [17], [20]. It has already been shown that sensors on the ankle support the recognition of ascending or descending stairs [26].

The range for the accelerometers was ±6 g. The range of the gyroscopes was ±500 deg/s for the sensor nodes wrist, chest, and hip and ±2000 deg/s for the sensor node on the ankle, since larger angular velocities are expected in the lower extremities. The sampling rate was set to 204.8 Hz and the data was stored on SD card.

**Figure 2:**
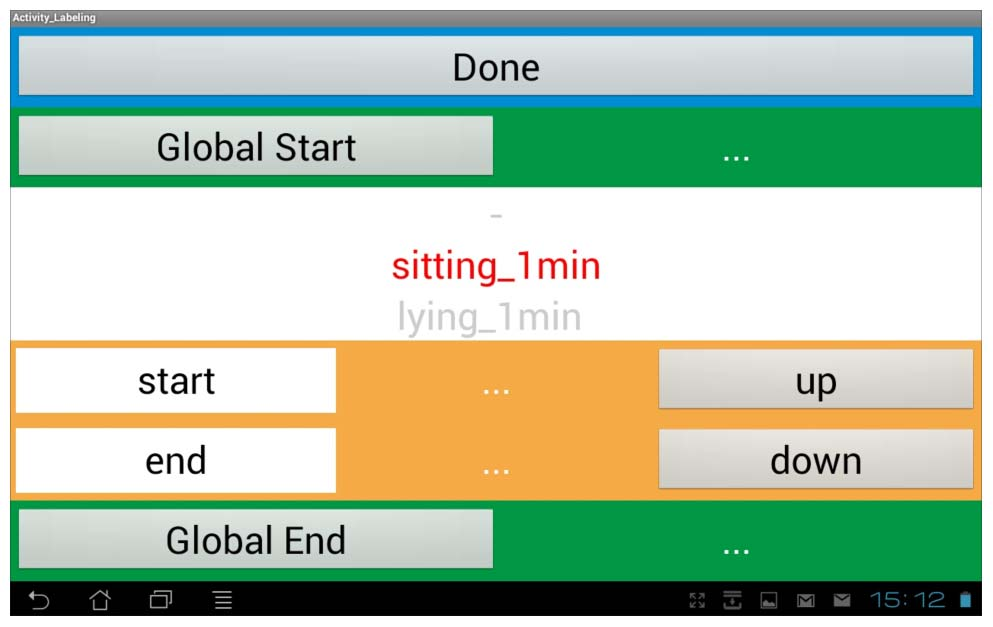
Screenshot of App used for data labeling purposes.

A mobile phone (Samsung Galaxy S2) was used as labeling device. An Android-based labeling App (Fig. 2) (running on the mobile phone) was used to label start time and end time of single activities concurrently to data collection.

The type of shirt and shoe (Fig. 1) was the same for all participants. We used four different shirt sizes (S, M, L, XL) in order to ensure tight fit and similar measurement conditions. To guarantee similar measurement conditions, we measured the chest width of each volunteer. Shirt sizes were assigned according to a size chart. The volunteers chose the shoe that they felt most comfortable in.

### Subjects

23 healthy subjects (10 female and 13 male, age 27±7 years, body mass index (BMI) 24.0 kg/m^2^±3.5 kg/m^2^, mean±standard deviation (SD)) were recruited for the study. Of these 23 subjects, 21 were right handed and two were left handed. The Research Ethics Committee of the Friedrich-Alexander-University Erlangen-Nuremberg confirmed that there is no necessity to obtain the approval of the local Ethics Committee. Ethics approval was deemed unnecessary because we measured only volunteers that were healthy, in good physical shape and did not suffer from a disease. All subjects gave written informed consent about their participation. All volunteers filled in the Physical Activity Readiness Questionnaire (PAR-Q [27]). The PAR-Q provides a self-administered screening before performing physical activity. The aim of the PAR-Q is to identify those people who should consult a doctor before performing physical activity. In the study, only those people who passed the PAR-Q were considered. The content of the PAR-Q can be found on http://www.activitynet.org. The study protocol involved 13 daily life activities that are normally performed every day. We used unobtrusive sensors that did not influence the volunteers and did not pose any additional risk to the volunteers. Furthermore, both supervisors of the study were first aid-trained. We did not conduct research outside our country of residence.

### Data Acquisition and Study Design

**Figure 3:**
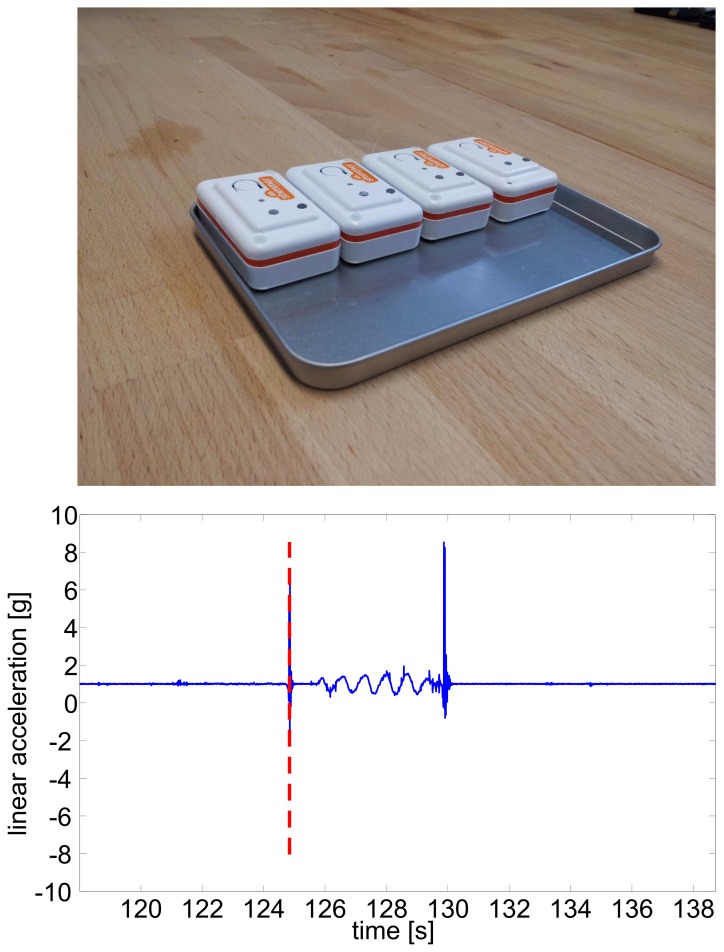
Sensor Synchronization. Plate with four SHIMMER sensor nodes used for synchronization (top) and sinusoidal synchronization signal (bottom). Dashed red line depicts the synchronization start point.

The subjects put the shoes, the T-shirt, the hip-clip, and the wrist band on (Fig. 1). The SHIMMER sensor nodes were powered on and put on a plate (Fig. 3, top). For offline synchronization, the plate was dropped down twice and, in between, the plate was moved up and down. The sensors were then placed on the dedicated measurement positions.

Subjects performed a total of 13 activities (Table 1), which were taken from the “Compendium of Physical Activity” published by Ainsworth et al. [28]–[30]. In these compendiums, physical activity is characterized in four categories depending on Metabolic Equivalent of Task (MET) values: sedentary (1.0–1.5 METs), light-intensity (1.6–2.9 METs), moderate-intensity (3.0–5.9 METs), and vigorous-intensity (≥6 METs) activities. Table 1 lists the durations and the intensities of the executed activities.

**Table 1:** List of studied activities, abbreviations, durations, and intensities expressed in Metabolic Equivalent of Task (MET).

Activity	Abbreviation	Duration [min]	Intensity [MET]
Sitting	SI	1	1.3
Lying	LY	1	1.0
Standing	ST	1	1.3
Washing dishes	WD	2	2.5
Vacuuming	VC	1	3.3
Sweeping	SW	1	3.3
Walking	WK	n.a.*	3.5
Ascending stairs	AS	n.a.**	5.0
Descending stairs	DS	n.a.**	3.5
Treadmill running	RU	2	9.0
Bicycling on ergometer (50 W)	BC50	2	3.5
Bicycling on ergometer (100 W)	BC100	2	6.8
Rope jumping	RJ	n.a.***	8.8

* All subjects had to walk on the university campus from one building to another building.

** All subjects had to climb stairs to the third floor and then back again.

*** All subjects had to perform 5 trials with at least 5 jumps each.

A researcher that labeled the start and end of each activity accompanied the subject during the whole data acquisition. First, the static activities (sitting, lying, standing) as well as the household activities were performed. The subject was told to use the vacuum cleaner with the right hand as this was the position for the wrist sensor. Otherwise, the wrist sensor signal delivered no suitable information about the signal pattern of the right hand. Then the subject had to walk on the university campus to another building. In this building, walking upstairs (until the third floor) and walking downstairs (back to the main floor) was recorded. Afterwards, the subject walked again on the university campus and performed the physically more demanding exercises indoor. One exercise included walking on a treadmill (h/p/cosmos quasar, h/p/cosmos & medical gmbh, Nussdorf-Traunstein, Germany). The treadmill speed was set to 8.3 km/h. Furthermore, the subjects had to bicycle on an ergometer (sanabike 250 F, MESA Medizintechnik GmbH, Benediktbeuern, Germany) with two different resistance levels (50 W and 100 W). The treadmill speed and the resistance level were chosen to obtain activities with different MET values (Table 1). The subjects were told to keep the revolutions per minute constant to 70 during the two different resistance levels. Thus, the differences of the two levels were not due to different revolutions per minute. Next, the subject had to perform the activity rope jumping. For this activity, the subject had to perform five trials with at least five jumps each.

**Figure 4:**
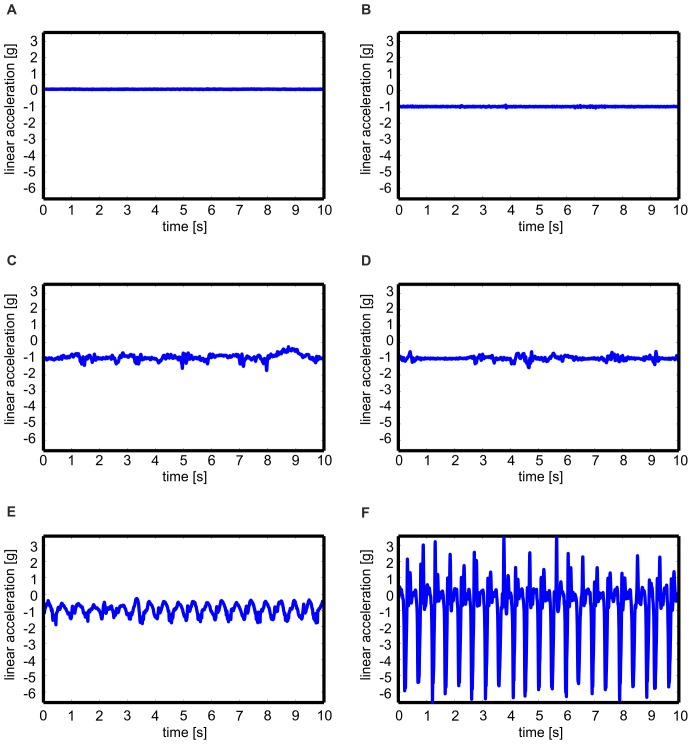
Example signals. Linear acceleration in vertical direction of the hip sensor for six activities. A: Lying, B: Standing, C: Vacuuming, D: Sweeping, E: Walking, F: Rope jumping.

As an example, Fig. 4 shows the linear acceleration in vertical direction of the hip sensor for the activities lying, standing, vacuuming, sweeping, walking, and rope jumping.

After the data acquisition, the SHIMMER sensor nodes were taken from the dedicated measurement positions and put again on the synchronization plate (Fig. 3). The described synchronization pattern was again performed in order to check if problems of the sensors occurred during the data acquisition. The SHIMMER sensor nodes were powered off and the kinematic data and the labeling data were stored on a PC for offline processing.

### Preprocessing

Four datasets had to be excluded from further processing. Of these four datasets, three datasets were excluded because of problems during gyroscope initialization. The fourth datasets was excluded as the data of the ankle sensor node was not available. In total, 19 datasets were used in the following.

The four SHIMMER sensor nodes were synchronized offline. For this, the first up-down movement of the sensor signal was manually selected in the linear acceleration of the vertical direction in all sensor nodes (Fig. 3, bottom, vertical line). This point constituted the common start point of all sensors.

The labeling was done automatically due to the saved start and end times of the Android app (Fig. 2). For each labeled activity, two seconds at the beginning and at the end were cut, in order to eliminate measuring errors during labeling.

### Proposed Classification System

**Figure 5:**
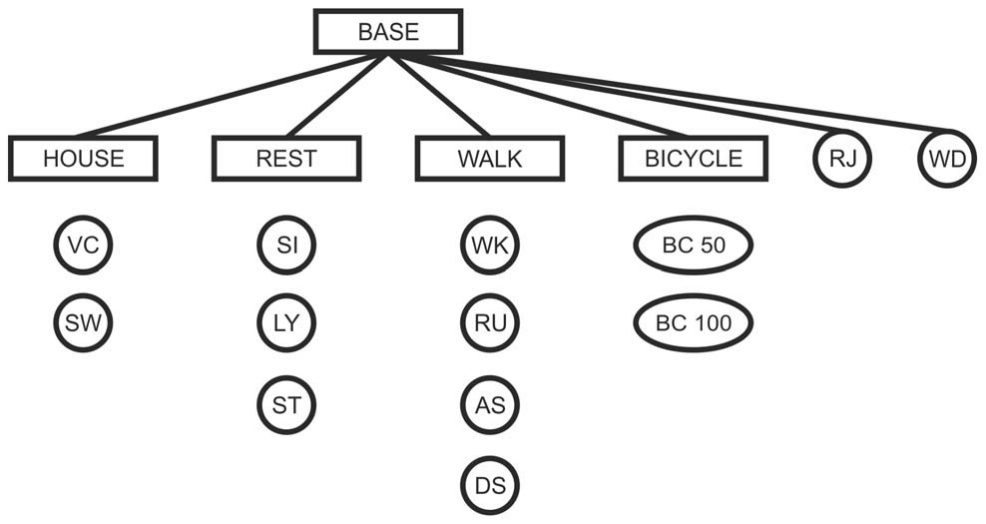
Illustration of the proposed classification system. Rectangles indicate single classification systems BASE, HOUSE, REST, WALK and BICYCLE. Circles indicate single activities VC (vacuuming), SW (sweeping), SI (sitting), LY (lying), ST (standing), WK (walking), RU (running), AS (ascending stairs), DS (descending stairs), BC 50 (bicycling, 50 watt), BC 100 (bicycling, 100 watt), RJ (rope jumping) and WD (washing dishes).

An overview of our proposed classification system is depicted in Fig. 5. The rectangles indicate single classification systems, whereas the circles indicate single activities. The general idea was to set up a hierarchy of classification systems, where each system solved a different classification problem. The first classifier in the hierarchy was the BASE classification system. It discriminated four activity groups and two single activities (rope jumping and washing dishes) that did not fit in any of the groups. The remaining four classifiers constituted the second hierarchy level. We chose such a hierarchical system, because new activities can be introduced without retraining all classifiers. The system was therefore flexible in its application to different activities.

### Preprocessing

The data processing was performed in sliding windows with 50% overlap [17], [20], [21]. The width of the window was set to 5 s, comparable to [17], [21], which used 6.7 s and 5.12 s, respectively.

### Feature Extraction

We defined a generic feature set for the classification systems BASE, HOUSE, WALK, and BICYCLE, which were computed for every sliding window. The generic feature set consisted of six features that were computed for every sensor axis and one feature that was computed for each of the accelerometer and gyroscope of each sensor node. The six features for every sensor axis included four time domain and two frequency domain features.

The four time domain features were:

- minimum amplitude
- maximum amplitude
- mean amplitude
- variance of amplitude.

The minimum and maximum amplitude extracted range information of the amplitude. The mean and variance of the amplitude gave important knowledge about statistics of the signal.

The two frequency domain features were:

- spectral centroid
- bandwidth.

Spectral centroid and bandwidth delivered important information about the frequency distribution of the activities [31].

The single feature that was computed for each sensor type (accelerometer or gyroscope) of one sensor node was the energy. The energy for each sensor type was calculated in three steps. First, the sum of the squared values for each axis was calculated. Second, the three sums were added together and divided by three. Third, this sum was divided by the number of samples. The energy gave important information about the activity level of a person. In total, this resulted in 152 features for each sliding window.

We defined a different feature set for the classification system REST. We extracted the gravitational component of the acceleration signal by a third-order elliptic low pass filter with an infinite impulse response and a cut-off frequency at 0.25 Hz [32]. This means that all three gravitational acceleration components of all four sensors were used as features. This was done because only the orientation of the body is important for the discrimination of the activities sitting, lying and standing. In total, this resulted in 12 features for each sliding window.

### Classification

Since there is no single classifier that is suitable for all classification tasks [33], the following classification systems were used [33], [34]: AdaBoost (ADA), classification and regression tree (CART), k-Nearest Neighbor classifier (kNN) and Support Vector Machine (SVM) with a radial basis function kernel. In the case of AdaBoost, 100 decision stump learners were used. In the case of kNN, k was set to five. The cost parameter of the SVM classifier was set to one and the gamma parameter to one divided by the number of features. For performance assessment, the mean class dependent classification rate and the overall mean classification rate were computed with a leave-one-subject-out procedure for all five classification systems. In each leave-one-subject-out trial, all epochs of one certain subject were removed from the training set. In order to evaluate the whole hierarchical classification system, the classifier with the best performance was chosen for the systems BASE, HOUSE, REST, WALK, and BICYCLE.

### Comparison to Algorithms in Literature

Our proposed method was compared to six state-of-the-art approaches in literature [17], [19]–[21], [23], [32]. We have chosen these approaches due to their citation rate and hence, their state-of-the-art research impact. Further, all six approaches had intersections with our study setup regarding the used sensors (accelerometers and gyroscopes), the sensor placement, and the performed activities.

An overview of the different approaches is shown in Table 2. The third column in the table shows according to each publication the kind of sensor and their original placement. We only used identical sensor positions and sensor data. This means that we only considered at maximum four sensor positions (Fig. 1) of accelerometer and gyroscope data and disregarded other signals like the heart rate. We modified, for example, the sensor placement and axes alignment suggested by Bao and Intille [17]. The authors used five biaxial accelerometers and placed them on the right hip, the right wrist, the left arm, the right ankle, and the left thigh. As we acquired data with triaxial accelerometers, we only used two axes for comparison and only the signals of the three sensors placed on the right hip, the right wrist, and the left ankle. These were the sensor positions for which we had identical placements compared to the work by Bao and Intille [17].

**Table 2:** Overview of six state-of-the-art approaches in the literature that were implemented and compared in the present study.

Year	Authors	# Sensor/placement	Used Sensor/placement	Samplingrate	Epoch duration	Used features	Best classifier	Evaluation process
2004	Bao and Intille[17]	5 biaxial accelerometers: right hip, right wrist, left arm, right ankle,left thigh	accelerometers: right hip, right wrist, left ankle	76.25 Hz	6.7 s (50% overlap)	mean, energy, frequencydomain entropy,correlation of theacceleration signals	decision tree classifier	leave-one-subject-out-cross-validation procedure
2005	Ravi et al. [21]	1 triaxial accelerometer: pelvic region	right hip	50 Hz	5.12 s (50% overlap)	mean, standard deviation,energy, correlation of theaccelerometer signals	plurality voting	10-fold cross-validation
2006	Karantonis et al.[23]	1 triaxial accelerometer: right hip	right hip	45 Hz	1 s (no overlap)	median filter, low passfilter, normalized signalmagnitude area, tilt angle	hierarchical, threshold based classifier	none
2006	Pärkkä et al. [23]	2 accelerometers: chest and wrist (dominant); additional signals like altitude, ECG,temperature,and heart rate	chest and right wrist	chest: 200 Hz; wrist: 40 Hz	1 s (no overlap)	peak frequency of up-down chest acceleration,median of up-down chestacceleration, peak powerof up-down chestacceleration, variance ofback-forth chestacceleration, sum ofvariances of three-dimensional wrist accelerations	automatically generated decision tree	leave-one-subject-out-cross-validation procedure
2009	Preece et al. [20]	3 triaxial accelerometers: waist, thigh, ankle	right hip, left ankle	64 Hz	2 s (50% overlap)	magnitude of first fivecomponents of FFTanalysis	kNN	leave-one-subject-out-cross-validation procedure
2012	Liu et al. [19]	2 triaxial accelerometers: hip (dominant hip) and wrist (dominant hand); ventilation sensor	right hip and right wrist	30 Hz	30 s (no overlap)	hip accelerometer: x-axis:standard deviation, 25thpercentile, and spectralentropy; y-axis: spectralentropy; z-axis: standarddeviation and 90thpercentile; wristaccelerometer: x-axis: 25th,50th, 75th 90th percentiles,standard deviation,spectral energy andentropy; y-axis: all time andfrequency domain features,z-axis: all time domainfeatures and spectral energy	SVM with radial basis function	leave-one-subject-out-cross-validation procedure

All six state-of-the-art approaches used a lower sampling frequency than our proposed sampling frequency of 204.8 Hz. In order to compare our method with the state-of-the-art approaches, the datasets were down-sampled to the sampling frequencies used in the corresponding approaches. The down-sampling was performed by a linear interpolation method. Furthermore, the epoch size was set according to the description of each publication. We implemented the features and the classifiers as described in the different approaches. We compared our method only to the suggested final feature set and the classifier with whom the best classification results were obtained. In order to use the same evaluation process in each approach, a leave-one-subject-out cross validation was performed in each of the six state-of-the-art approaches, except in the algorithm of Karantonis et al. [32]. Karantonis et al. did not apply a training step and used predefined and fixed thresholds.

Each approach in literature used different activities. We evaluated all six approaches on all recorded activities in our work, except for the algorithm of Karantonis et al. [32]. The hierarchical, threshold based classifier used in Karantonis et al. was optimized for fall detection and therefore not applicable for all recorded activities in our work. For the other five approaches, this means, that we investigated activities that were not considered in the original works.

## Results

Table 3 shows the overall mean classification rates after leave-one-subject-out procedure. AdaBoost was the best classifier for the HOUSE system. kNN was the best classifier for the WALK system. SVM was the best classifier for the BASE, REST and BICYCLE system. Table 4 shows the mean class dependent classification rates and the overall mean classification rates of our proposed and the compared algorithms [17], [19]–[21], [23], [32]. The confusion matrix of our proposed algorithm can be seen in Table 5.

**Table 3:** Mean classification rates (in percent) of the five subsystems (BASE, REST, HOUSE, WALK, BICYCLE).

	ADA	CART	kNN	SVM
**BASE**	64.8	96.1	97.7	**97.9**
**HOUSE**	**89.9**	84.0	86.5	85.5
**REST**	95.1	92.7	96.1	**97.4**
**WALK**	94.7	93.3	**97.7**	94.3
**BICYCLE**	60.8	53.7	49.4	**61.6**

Best results are printed bold.

**Table 4:** Mean class dependent classification rates (in percent) for all 13 activities and overall mean classification rates of proposed system and state-of-the-art systems [17], [19]–[21], [23], [32].

	[17]	[21]	[32]	[23]	[20]	[19]	Proposed
**SI**	83.1	83.7	81.8	65.5	67.7	38.1	**88.9**
**LY**	94.5	87.6	94.7	98.2	**100.0**	57.4	**100.0**
**ST**	80.7	60.2	64.0	65.6	48.6	44.4	**89.8**
**WD**	88.9	79.0	–*	75.4	56.0	89.6	**98.1**
**VC**	66.9	17.7	–*	36.0	39.2	42.9	**85.4**
**SW**	81.2	57.8	–*	60.7	62.6	54.3	**89.9**
**WK**	96.2	93.0	98.7	74.3	97.6	88.1	**99.0**
**AS**	79.5	18.6	–*	28.5	70.6	29.3	**95.5**
**DS**	73.1	16.4	–*	44.2	60.2	35.3	**95.2**
**RU**	**100.0**	98.3	–*	92.7	97.2	94.4	**100.0**
**BC50**	48.1	50.4	–*	40.2	64.3	47.8	**69.1**
**BC100**	48.5	11.7	–*	46.2	41.7	48.3	**53.5**
**RJ**	99.4	93.4	–*	76.6	86.7	33.3	**100.0**
**mean**	80.0	59.1	84.8	61.8	68.7	54.1	**89.6**

Best results are printed bold.

* The algorithm of (Karantonis et al. [32]) was not applied to all activities, as activity optimized features were used.

**Table 5:** Confusion matrix of our proposed algorithm. Each entry represents the number of classified epochs.

	SI	LY	ST	WD	VC	SW	WK	AS	DS	RU	BC50	BC100	RJ
**SI**	**383**	0	22	14	1	2	0	0	2	0	7	0	0
**LY**	0	**435**	0	0	0	0	0	0	0	0	0	0	0
**ST**	0	0	**386**	27	9	0	0	0	0	0	8	0	0
**WD**	0	0	10	**896**	5	1	0	0	0	0	1	0	0
**VC**	0	0	0	0	**369**	55	0	0	0	0	6	2	0
**SW**	0	0	1	6	43	**643**	4	7	5	0	4	2	0
**WK**	0	0	0	0	4	4	**1995**	5	7	0	0	0	0
**AS**	0	0	0	0	0	3	10	**279**	0	0	0	0	0
**DS**	0	0	0	0	0	1	11	0	**237**	0	0	0	0
**RU**	0	0	0	0	0	0	0	0	0	**886**	0	0	0
**BC50**	0	0	0	0	4	22	0	1	0	0	**620**	250	0
**BC100**	0	0	0	1	0	6	0	0	0	0	410	**480**	0
**RJ**	0	0	0	0	0	0	0	0	0	0	0	0	**236**

The confusion matrices of each leave-one-subject-out trial were summed up.

## Discussion

In this paper, we developed a hierarchical classification system that was able to distinguish between 13 DLAs. Further, we compared our proposed method to six state-of-the-art approaches in the literature [17], [19]–[21], [23], [32]. In the following, these two aspects of this study are discussed in detail.

### Subsystems BASE, REST, HOUSE, WALK, and BICYCLE

We divided our hierarchical classification system into five subsystems. The BASE system is the basis for the differentiation in the four subsystems REST, HOUSE, WALK, and BICYCLE and the two activities rope jumping and washing dishes.

The best classifier for the BASE system was SVM with an overall mean classification rate of 97.9% (Table 3). SVM is known as a classifier with a good generalization performance [34]. The overall mean classification rate of the AdaBoost classifier was rather low compared to CART, kNN and SVM. The reason was the low mean class dependent classification rate of the two single activities washing dishes and rope jumping, which heavily decreased the overall mean classification rate of the AdaBoost classifier. The number of learners seemed to be too low for this classification problem. Further research using these activities might take this into account and increase the number of learners.

All in all, the high maximum overall mean classification rate of 97.9% showed the applicability of the BASE system to distinguish between activity groups and single activities. The grouping provided the possibility to use different classifier types for different groups of activities. This enhanced the flexibility of the classification system.

The overall mean classification rates of the REST system ranged from 92.7% to 97.4% (Table 3). Thus, all classifiers were suitable for the distinction between the static activities. Furthermore, the results showed that the reduced feature set was suitable for this classification task. The best overall mean classification rate was obtained with the SVM classifier.

The overall mean classification rates of the classifiers for the HOUSE system ranged from 84.0% to 89.9% (Table 3). Although the signal patterns of vacuuming and sweeping are similar (Fig. 4), the proposed feature set was suitable to distinguish between these two activities. The best classifier of the HOUSE system was AdaBoost. The reason might be that AdaBoost is an ensemble system, which reduces the variance and increases the confidence of the classifier decision.

The kNN classifier was the best classifier in the case of the WALK system and reached an overall mean classification rate of 97.7% (Table 3). Thus, walking patterns at different inclinations and speed levels can be distinguished. It is assumed that the gyroscope of the ankle provides useful information about the inclination, which is also stated in [26]. In order to further improve the performance of the hierarchical system, the activities walking and running can be grouped, as well as descending and ascending stairs. The corresponding two new subsystems can be added in our proposed system after the WALK system (Fig. 5).

The overall mean classification rates of all classifiers in the case of the BICYCLE system were rather low compared to the classification rates of the other four subsystems (Table 3). Since the revolutions per minute were kept constant, it was hard to distinguish between the two resistance levels. The best classifier was the SVM, which reached an overall classification rate of 61.6% (Table 3).

All in all, the results (Table 3) showed that different classifier types achieved the best overall mean classification rate regarding each of the five subsystems BASE, HOUSE, REST, WALK and BICYCLE. SVM was chosen three of five times as the best classifier due to the known good generalization performance. As AdaBoost and kNN achieved better results in two subsystems, applying different classifier types for different groups of activities is therefore mandatory. This endorses that no single classifier is suitable for all classification tasks [33].

### Comparison of Proposed System to State-of-the-art Algorithms in Literature

We compared our hierarchical classification system with six state-of-the-art algorithms in literature [17], [19]–[21], [23], [32].

The algorithm described by Bao and Intille [17] reached an overall mean classification rate of 80.0% (Table 4). The mean class dependent classification rates of sitting, standing, and lying (Table 4) were smaller compared to the given classification rates in [17]. Especially, the mean class dependent classification rates of sitting and standing were considerably higher (94.8% and 95.7% in [17] compared to 80.7% and 83.1%). It is assumed that the additional sensor on the thigh increased the mean classification rates in [17]. On the other side, the mean class dependent classification rates of walking and running were higher using our dataset (89.7% and 87.7% in [17] compared to 96.2% and 100.0%). This might be due to the different sensor position at the lower limb.

The algorithm described by Ravi et al. [21] reached an overall mean classification rate of 59.1% (Table 4). The reason might be that only one sensor on the hip was used. Therefore, the classification of activities including upper and lower extremity motions was challenging. This was indicated by the rather low mean class dependent classification rates of the activities vacuuming, sweeping, ascending stairs, descending stairs, and bicycling.

The algorithm described by Karantonis et al. [32] reached the best mean class dependent classification rate for walking (Table 4). The reason is that besides the detection of the postural orientation (tilt angle feature), Karantonis et al. used an optimized algorithm for walking. All used features were matched to their performed activities (mainly transitions between standing, lying, and sitting and different fall scenarios). The mean class dependent classification rates of sitting and standing were rather low compared to lying and walking (Table 4). It is assumed that instances of sitting were misclassified as standing and vice versa, which was also mentioned in [32]. The focus of Karantonis et al. was to detect possible falls and hence, this misclassification is not severe. Their focus was a real-time implementation for ambulatory monitoring. Their algorithm could only be applied to a subset of our recorded activities. Therefore, it is difficult to compare the overall performance to the other approaches and our proposed algorithm.

The algorithm described by Pärkkä et al. [23] reached a rather low overall mean classification rate of 61.8% compared to the other algorithms (Table 4). The reason might be that only two sensors (one sensor on the chest and one sensor on the wrist) were used. Therefore, as mentioned before, the classification of activities including lower extremity motions was challenging. This is indicated by the low mean class dependent classification rates of ascending stairs, descending stairs, and bicycling. Although vacuuming includes motions that should be recognized by the chest and wrist sensor, the mean class dependent classification rate was low. It is assumed that instances of vacuuming were misclassified as sweeping, whose signal patterns are similar to signal patterns of vacuuming (Fig. 4). Regarding the discrimination of the static activities, the mean class dependent classification rate of lying was rather high compared to sitting and standing. The reason might be that Pärkkä et al. merged sitting and standing to one class. Thus, the used sensor placements were not able to distinguish between these two activities.

The algorithm described by Preece et al. [20] reached the overall mean classification rate of 68.7% (Table 4). Preece et al. focused on the comparison of different feature sets optimized for dynamic activities. They implemented wavelet features of five separate studies [35]–[39], proposed two own wavelet feature sets and compared each wavelet set to seven time and frequency domain feature sets. Preece et al. obtained the best result with the feature set of the magnitudes of the first five components of FFT analysis (Table 2). This feature set was selected due to dynamic activities. This might be the reason that the static activities sitting and standing were classified with only 67.7% and 48.6%, respectively. Preece et al. did not perform a feature selection as they wanted to compare different feature sets. The results might increase, if all features (wavelet, time and frequency domain features) were combined in one feature set and an appropriate feature selection procedure was applied to this feature set before the classification process.

The algorithm described by Liu et al. [19] reached the overall mean classification rate of 54.1% (Table 4). Liu et al. used the epoch size of 30 s. This epoch size is not compatible with the duration of our recorded activities. For the activity rope jumping, the subjects had to perform five trials with at least five jumps each. Hence, the duration of this activity was not always 30 s long. Consequently, some rope jumping datasets were not used in the classification process which yielded a low classification result of 33.3%. Five of our recorded activities lasted for only one minute (Table 1). Since two seconds were cut at the beginning and at the end of the labeled activities, classification of these five activities was based on only one epoch, which might not be enough for robust classification. The activities were correctly classified with mean class dependent classification rates from 38.1% to 54.3%. We included three household activities in our study setup. Two of them were only recorded for one minute, and hence, classification rates of only 42.9% and 54.3% (Table 4) were obtained. The third household activity (washing dishes) was classified with 89.6%. This might be because this activity was performed for two minutes (Table 1), which might lead to better performance in [19]. Liu et al. [19] performed a two-step feature selection. First, they performed a statistical analysis that was followed by the minimal-redundancy-maximal-relevance heuristic [40]. This resulted in a specialized feature set (Table 2). The problem with specialized feature sets is that they might not be applicable to all activities. This might be the reason why only three activities were classified with higher than 80% and activities that were not considered in the original study setup [19] like ascending and descending stairs were classified with low classification rates of 29.3% and 35.3%, respectively.

Our proposed method reached the overall mean classification rate of 89.6% (Table 4). It is assumed that different number of sensors due to different study setups and different identical sensor positions influenced the results. We used four sensor positions and compared these four sensor positions to one sensor position [21], [32], two sensor positions [20], [23], and three sensor positions [17], [19]. Thus, by using more sensors more complex activities can be classified.

We suggest to use sensors near the body’s center of mass (hip and chest) in order to cover a wide range of basic activities such as sitting, standing, lying, and walking. Nevertheless, the mean class dependent classification rates of sitting and standing were smaller compared to lying (Table 4). This trend coincides with the results of the other approaches in Table 4. It is assumed that an additional sensor on the thigh improves the performance of the classification system.

Moreover, we suggested to use sensors on lower and upper extremities (wrist and ankle) to distinguish between more complex activities like ascending stairs, descending stairs, vacuuming, and sweeping. Nevertheless, vacuuming was often misclassified as sweeping and vice versa (Table 5). In this case, an additional sensor on the other wrist might incorporate additional information into the classification system. Descending and ascending stairs were often misclassified as walking (Table 5). In order to reduce the misclassification, specialized gait features might improve the results for the WALK system. Nevertheless, given the flexibility of the proposed classification system, the incorporation of these ideas is straightforward.

The high mean class dependent classification rates of washing dishes (98.1%) and rope jumping (100.0%) (Table 4) showed again the applicability of the BASE system to distinguish between single activities and activity groups.

The high overall mean classification rate of the BASE system (Table 3) showed that it is possible to classify the merged activity group of the two resistance levels of bicycling. The rather low mean class dependent classification rates of the two resistance levels of bicycling 69.1% and 53.5% (Table 4) showed the challenge to distinguish between the single resistance levels. The reason is that the low resistance level was misclassified as the high resistance level and vice versa, which is confirmed by observations in the confusion matrix. Bicycling with the lower resistance level (50 W) was correctly classified in 620 cases and misclassified as bicycling with the higher resistance level (100 W) in 250 cases (Table 5). Bicycling with the higher resistance level was correctly classified in 480 cases and misclassified as bicycling with the lower resistance level in 410 cases.

The algorithm described by Bao and Intille [17] was the best result that we obtained with an algorithm used for comparison that we applied to all performed activities. The reasons might be the following:

- The study design of Bao and Intille and our study design had three sensor positions in common. In the other approaches, at maximum two sensor positions were identical.
- The approach by Bao and Intille was applied on twenty activities under both controlled conditions in the laboratory and semi-naturalistic conditions outside the laboratory. Thus, the approach by Bao and Intille seemed to be applicable for a large set of DLAs, especially under semi-naturalistic conditions.

Compared to Bao and Intille, we only collected data under controlled conditions. Thus, data under more realistic conditions would be desirable and it is planned to integrate these more realistic conditions in our work.

However, our proposed method showed that the classification of DLAs can benefit from sensor data fusion of accelerometer and gyroscope (Table 4). It is assumed that especially the gyroscope improves the classification of activities which include rotational movements like washing dishes, ascending stairs or rope jumping. Most approaches found in literature only used accelerometers. Nevertheless, Lee and Mase [24] and Najafi et al. [41] used the combination of accelerometer and gyroscope for the classification of body postures and walking behaviors like ascending stairs or walking. However, the classification systems were optimized for a certain subset of activities and cannot be applied to the recorded activities in our work. Koskimaki et al. [42] used the combination of accelerometer and gyroscope for the classification of activities of workers on industrial assembly lines. Altun and Barshan [43] used the combination of accelerometer and gyroscope for the classification of nineteen DLAs. Koskimaki et al. [42] and Altun and Barshan [43] computed features which were not optimized for a certain subset of activities and therefore, it is planned to compare our approach with [42] and [43], too.

For the performance assessment, a leave-one-subject-out procedure was applied. This procedure results in a small bias and a large variance of the true error rate estimator [44]. Due to the sample size, the leave-one-subject-out procedure was preferred to for example a 10-fold cross-validation, which shows a good performance for a larger sample size.

Since, multiple subjects perform multiple activities in different ways, there might be a high intersubject variability. In order to setup a generalized system that shows good performance for an unknown subject, the classification systems were trained based on multiple subjects.

All in all, our hierarchical, multi-sensor based classification system had problems in the distinction of the different activities of the HOUSE and BICYCLE systems. Both systems have to be improved. The activities sitting and lying were classified with mean class dependent classification rates below 90%. An additional sensor on the thigh could increase these classification rates. We calculated 152 features for each sliding window for each classification system (except for the REST system). A high number of features leads to high computational complexity in real-time applications or in embedded systems. Hence, an automatic reduction of this feature set like sequential forward selection [34] should be applied. Nevertheless, the overall mean classification rate of 89.6% showed the applicability of our proposed system to classify the acquired 13 activities.

The hierarchical structure of our proposed system has four advantages:

- Different classifiers can be used for the classification of different activity groups.
- Additional activities can easily be integrated without retraining the complete system.
- In many applications, in which the further classification of the activities in the activity groups is not necessary, the HOUSE, REST, WALK, and BICYCLE system can easily be neglected.
- A different window size can be chosen for the BASE, HOUSE, REST, WALK, and BICYCLE system, which might increase the classification rate.

## Conclusion

Physically inactive people have to be motivated to be more active so that their risk of various chronic diseases will decrease. A first step is to provide feedback about the individual quality of life. In this field, the classification of DLAs is of major interest.

In this paper, a novel, hierarchical, multi-sensor based classification system was developed, which reached an over all mean classification rate of 85.8%. We considered the classification of 13 DLAs. Furthermore, our proposed system was compared to state-of-the-art algorithms in literature using the same dataset. The comparison showed that the proposed data fusion of accelerometer and gyroscope provided a useful tool to distinguish between complex activities like ascending stairs or descending stairs.

A multitude of activity classification systems has been proposed in literature, and to date it is not clear which solution is outperforming the others and is applicable to a variety of real world scenarios. It is mandatory for the community to provide benchmark datasets and reference implementations. This will help to speed up the process of getting the best performing and most appropriate DLA classification system into much needed real world applications. We are inviting fellow scientists to share their data and implementations on our newly erected internet platform (http://www.activitynet.org).
